# Mineral-specific effects on humification and bacterial community succession in industrial-scale spent mushroom substrate composting

**DOI:** 10.3389/fmicb.2026.1952248

**Published:** 2026-09-04

**Authors:** Yuan Chang, Nannan Miao, Yuhan Zhang, Yuquan Wei, Yanbin Guo, Yizhi Sheng, Jing Xie, Licheng Zhang, Ji Li

**Affiliations:** 1 College of Resources and Environmental Sciences, China Agricultural University, Beijing, China; 2 Organic Recycling Institute (Suzhou) of China Agricultural University, Suzhou, China; 3 Center for Geomicrobiology and Biogeochemistry Research, State Key Laboratory of Biogeology and Environmental Geology, China University of Geosciences, Beijing, China; 4 Genliduo Bio-Tech Corporation Ltd., Xingtai, China

**Keywords:** compost humification, core bacterial genera, dissolved organic matter, mineral amendment, spent mushroom substrate

## Abstract

Spent mushroom substrate (SMS) is a lignocellulose-rich agricultural by-product whose slow humification can limit the quality and agronomic value of the resulting compost. This study compared four low-cost mineral amendments: shale powder (S), mullite-based soil conditioner (SC), flue-gas desulfurization gypsum (DG), and rare-earth silico-titanate ore powder (TO). Each mineral was added at 10% of SMS dry mass during 40-day industrial-scale composting. Mineral structure, physicochemical properties, humic fractions, dissolved organic matter (DOM) fluorescence components, and bacterial succession were evaluated. Results showed that mineral amendments accelerated pile heating, prolonged the thermophilic phase, and promoted compost humification. Relative to CK, final humic acid (HA) content increased by 20.6, 28.1, 10.7, and 7.9% in S, SC, DG, and TO, respectively, while the humification index (HI) increased by 25.9, 41.5, 14.9, and 16.0%, respectively, highlighting SC as the strongest overall humification response. EEM-PARAFAC analysis revealed mineral-specific DOM transformation patterns: the relative abundance of the highly humified C3 component increased by 9.1 and 17.3% in S and SC, respectively, whereas DG preferentially increased C2 by 18.8%, and TO caused a slight 2.3% decrease in C3 relative to CK. SC increased the relative proportion of the highly humified C3 component by 17.3%, whereas DG increased that of C2 by 18.8% relative to CK. Mineral amendments also reshaped bacterial succession in a stage-dependent manner. SC favored heat-adapted core genera associated with organic matter transformation and humification during the thermophilic and cooling phases, whereas DG and TO exerted stronger selection for maturation-associated Actinomycetota, particularly *Nonomuraea*. Mantel analysis further revealed significant associations between mineral-induced shifts in the core bacterial community and changes in composting conditions and humification characteristics (Mantel *r* = 0.323–0.728, FDR-adjusted *p* < 0.05). These findings demonstrate that mullite-based conditioner provided the most effective enhancement of SMS compost maturity and humification, providing a scientific basis for the targeted selection of low-cost mineral amendments and the valorization of agricultural by-product.

## 1 Introduction

Agricultural production generates large quantities of organic by-products that retain substantial amounts of carbon and nutrients but can become environmental burdens when they are not effectively recycled (Babu et al., 2022). Spent mushroom substrate (SMS), the residual growth medium remaining after mushroom cultivation, is one such rapidly increasing resource. Approximately 5 kg of SMS is generated per kilogram of fresh mushrooms, and China contributes more than 70% of global mushroom production (Khan et al., 2026; Ma et al., 2025). Composting offers a practical route for converting SMS into a stable organic amendment by coupling microbial decomposition with the formation of humic substances. However, SMS contains abundant lignocellulose and structurally complex aromatic compounds, which can slow organic-matter turnover and constrain humification (Ma et al., 2025). Improving the conversion of these recalcitrant components into stable humic fractions is therefore important for increasing both the fertilizer value and carbon retention potential of SMS compost.

Mineral amendments have received increasing attention as functional regulators of composting because their effects extend beyond simple bulking. Their pore networks and surface area can modify pile aeration, moisture distribution, and microsites for microbial colonization, whereas surface functional groups and ion-exchange sites can retain dissolved organic matter (DOM), ammonium, and other reactive constituents (Huang et al., 2022; Muscarella et al., 2021). These interfacial processes may reduce the loss of humic precursors and bring organic molecules, microorganisms, and reactive mineral surfaces into closer contact. Minerals may also influence humus formation through surface complexation, cation bridging, and catalytic reactions that alter the balance between biotic transformation and abiotic condensation (Chen et al., 2023). Minerals may therefore influence humification through multiple pathways, including improvement of the composting microenvironment, regulation of microbial processes, and promotion of abiotic polymerization. Recent adsorption experiments further demonstrated that different minerals exhibit distinct capacities for the selective adsorption and retention of humic acid (HA), fulvic acid (FA), and other DOM components (Jiao et al., 2025). These findings suggest that variations in mineral structure and chemical composition may differentially affect organic matter decomposition, humus formation, and microbial community succession, thereby resulting in mineral-specific effects on the composting process.

Most composting studies have focused on individual natural minerals or closely related clay minerals. Zeolite can improve nitrogen retention (Wang et al., 2024), sepiolite can enhance organic-matter transformation and reduce metal bioavailability (Zheng et al., 2022), and bentonite or attapulgite can promote compost maturation and HA formation (Cao et al., 2025; Zhang and Sun, 2017). Although these studies demonstrate the functional potential of mineral amendments, side-by-side industrial-scale evidence comparing natural, synthetic, and mineral-derived industrial by-product amendments remains scarce. Compared with conventional natural minerals, synthetic minerals offer controllable structures and stable performance, whereas mineral-derived industrial by-products provide additional advantages, including wide availability, low cost, and high resource-recovery potential (Brunchi and Morariu, 2024; Rangappa et al., 2024). However, how the intrinsic properties of different minerals are translated into distinct humification outcomes remains poorly understood. In particular, it is unclear how differences in pore architecture and interfacial chemistry influence the retention and transformation of specific DOM fractions, and whether these physicochemical constraints are accompanied by distinct stage-dependent bacterial succession. Consequently, a mechanistic link connecting mineral properties, DOM transformation, microbial succession, and the resulting humification response has yet to be established.

This study selected four low-cost materials with resource-recovery potential-natural shale, a synthetic mullite-based conditioner, flue-gas desulfurization gypsum, and rare-earth silico-titanate ore powder-to compare their effects on industrial-scale SMS composting. Physicochemical parameters, humic fractions, DOM fluorescence components, and bacterial succession were jointly evaluated. We hypothesized that contrasting mineral physicochemical properties would generate mineral-specific humification trajectories through the coupling of abiotic mineral-organic interactions and stage-dependent bacterial succession. This study aimed to reveal the differential humification effects of minerals with distinct origins and properties and to provide a scientific basis for developing low-cost functional mineral amendments and promoting the synergistic valorization of agricultural wastes and mineral resources.

## 2 Materials and methods

### 2.1 Composting feedstock and mineral amendments

Spent mushroom substrate (SMS), the solid residue remaining after mushroom cultivation, was obtained from Linxi Jiudaogu in Hebei Province, China. Four mineral amendments were evaluated: shale powder (S), mullite-based soil conditioner (SC), flue-gas desulfurization gypsum (DG), and rare-earth silico-titanate ore powder (TO). Shale, desulfurization gypsum, and rare-earth silicon–titanium ore powder were obtained from suppliers in Zhangjiakou, Hebei Province. The soil conditioner was supplied by Genliduo Bio-Tech Corporation Ltd., Xingtai, Hebei Province, China. The initial physicochemical properties of the SMS and minerals are reported in Supplementary Table S1. The initial properties of the prepared composting mixtures are provided in Supplementary Table S2.

### 2.2 Industrial-scale trough composting

Industrial-scale composting was conducted at the production facility of Genliduo Biotechnology Co., Ltd., Wei County, Xingtai, Hebei Province, China. Five treatments were established: an unamended control (CK) and four mineral-amended treatments (S, SC, DG, and TO). Each mineral amendment was incorporated at 10% of the SMS dry mass, and the initial moisture content of all mixtures was adjusted to approximately 60%. Five spatially separated windrows were established in two fermentation troughs (63 × 5 × 1.2 m), with one windrow assigned to each treatment. Each windrow was considered the experimental unit; therefore, each treatment consisted of one independent windrow (treatment-level *n* = 1). The five treatment windrows were distributed between the two fermentation troughs according to operational constraints at the industrial production site. In trough 1, the windrows were arranged from left to right as CK, SC, and S, whereas trough 2 contained DG and TO. Windrows located within the same trough were separated by approximately 3 m. The composting materials were loaded using a forklift and turned periodically using a mechanical compost turner. Windrow height was maintained at approximately 1.0–1.2 m, and the ambient temperature inside the fermentation workshop ranged from 35 to 44 °C throughout the composting period.

Compost samples were collected on days 0, 4, 7, 14, 18, 31, and 40. At each sampling time, spatial subsamples were collected from the front, middle, and rear sections of each windrow and thoroughly homogenized to obtain one representative composite sample (approximately 500 g) per windrow. These spatial subsamples were collected to improve the representativeness of within-windrow sampling and were not treated as independent experimental replicates. Each homogenized composite sample was divided into two portions. One portion was stored at 4 °C for physicochemical and humification-related analyses, whereas the other was stored at −80 °C for subsequent DNA extraction.

Four representative composting stages were selected for microbial community analysis: day 0 (heating phase, H), day 18 (thermophilic phase, T), day 31 (cooling phase, C), and day 40 (maturation phase, M).

### 2.3 Mineral characterization and physicochemical analyses

Mineral morphology was examined by scanning electron microscopy (GeminiSEM 300, ZEISS, Germany). Specific surface area was determined using the Brunauer–Emmett–Teller (BET) method with an automated surface-area and porosity analyzer (ASAP 2460, Micromeritics, USA).

Temperature was measured daily at multiple positions within each windrow using insertion-probe thermometers. The pH, electrical conductivity (EC), moisture content, and germination index (GI) of the compost samples were determined according to the Chinese Organic Fertilizer Standard (NY/T 525–2021). Total organic carbon (TOC) and total nitrogen (TN) were determined using a CHNS elemental analyzer.

### 2.4 Humus extraction and DOM EEM-PARAFAC analysis

Humic substances (HS), including humic acid (HA) and fulvic acid (FA), were extracted following the method described in Chang et al. (2023). The carbon concentrations of HS, HA and FA were determined using an organic carbon analyzer (TOC-L CPH, Shimadzu, Japan). The humification indices were calculated following the formulas described by Yang et al. (2023).

$$\text{Humification index}(\mathrm{HI})=\frac{C_{\mathrm{HA}}}{\mathrm{TOC}}\times 100$$

$$\text{Humification Ratio}(\mathrm{HR})=\frac{C_{\mathrm{HS}}}{\mathrm{TOC}}\times 100$$

$$\text{Degree of polymerization}(\mathrm{DP})=\frac{C_{\mathrm{HA}}}{C_{\mathrm{FA}}}$$

C_HA_, C_HS_, and C_FA_ represent the carbon contents of humic acid (HA), humic substances (HS), and fulvic acid (FA), respectively, and TOC represents the total organic carbon content of the compost. All TOC, TN, HS, HA, and FA values reported in this study were expressed on a dry-mass basis of the mineral-amended compost samples.

Dissolved organic matter (DOM) was extracted from fresh compost samples using deionized water at a solid-to-liquid ratio of 1:10 (w/v) on a dry-mass-equivalent basis, followed by shaking and filtration through a 0.45-μm membrane filter. Excitation-emission matrices (EEMs) of DOM were measured using a fluorescence spectrophotometer (F-7000, Hitachi, Japan), with excitation and emission wavelengths ranging from 200 to 600 nm. The excitation and emission wavelength intervals were 10 and 5 nm, respectively, and the scanning speed was 12,000 nm min^−1^. Corresponding Milli-Q water blanks were subtracted from the sample EEMs to correct for background fluorescence. Inner-filter effects were corrected prior to PARAFAC analysis. Fluorescence intensities were subsequently normalized to Raman units using the integrated Raman peak of Milli-Q water at an excitation wavelength of 350 nm. Regions affected by Rayleigh and Raman scattering were removed prior to PARAFAC modeling.

PARAFAC analysis was performed in MATLAB R2024b using the DOMFluor toolbox. Candidate models containing two to six fluorescent components were evaluated. Model selection and validation were based on the examination of excitation and emission spectral loadings, model residuals, sum-of-squared errors, core consistency, split-half validation, and repeated random initialization.

### 2.5 DNA extraction and high-throughput sequencing

Total genomic DNA was extracted from compost samples collected at the heating, thermophilic, cooling, and maturation stages using the FastDNA Spin Kit for Soil (MP Biomedicals, USA). The V3-V4 region of the bacterial 16S rRNA gene was amplified using primers 338F (5′-ACTCCTACGGGAGGCAGCAG-3′) and 806R (5′-GGACTACHVGGGTWTCTAAT-3′). PCR products were verified by 2% agarose-gel electrophoresis, quantified using a QuantiFluor-ST Blue system, and used to construct TruSeq libraries. Paired-end sequencing was performed in PE300 mode on an Illumina platform by Majorbio Bio-Pharm Technology Co., Ltd. (Shanghai, China).

Raw reads were quality-filtered using fastp v0.23.4 and merged using FLASH v1.2.11. Reads shorter than 50 bp, containing more than five ambiguous bases, or with a mean quality score below 20 within a 50-bp sliding window were discarded; paired-end reads were merged with a minimum overlap of 10 bp and a maximum mismatch ratio of 0.2. A total of 1,080,333 raw sequences were generated across all sequenced samples, of which 1,063,602 valid sequences were retained after quality filtering and merging (98.45%), with 44,450–65,111 valid sequences obtained per sample. OTUs were clustered at 97% sequence identity using UPARSE in USEARCH v11, with chimeric sequences removed. Taxonomic annotation was performed using the RDP Classifier v2.11 against the SILVA 138.2 bacterial 16S rRNA database at a confidence threshold of 70%, followed by removal of chloroplast and mitochondrial sequences. The resulting OTU table contained 980,088 sequences and was rarefied to 40,721 sequences per sample, yielding 1,596 OTUs for downstream analyses. At the phylum and genus levels, 98.93 and 59.15% of OTUs were assigned to named taxa, respectively, accounting for 99.98 and 92.83% of the rarefied sequences.

Microbial co-occurrence networks were constructed at the OTU level based on Spearman rank correlations of relative-abundance data. To reduce the influence of rare taxa and improve network robustness, OTUs with a mean relative abundance ≤0.05% or occurring in <20% of the samples were excluded prior to network construction. Pairwise Spearman correlations were calculated among the retained OTUs using the corr.test function in the R package psych. *p* values were adjusted for multiple testing using the false discovery rate (FDR) procedure. Only associations with an absolute Spearman correlation coefficient (|*r*|) ≥ 0.6 and an FDR-adjusted *p* value ≤ 0.05 were retained as network edges. Positive and negative coefficients were interpreted as positive and negative co-occurrence associations, respectively.

### 2.6 Statistical analysis

All physicochemical, humification, and fluorescence-related parameters were measured in triplicate using aliquots of the same homogenized composite sample. Results are presented as the mean ± standard deviation (SD) of the three analytical determinations. These triplicate measurements represent analytical replication used to assess analytical repeatability and were not considered independent experimental replicates. Differences among treatments were evaluated using one-way analysis of variance (ANOVA), followed by Tukey’s honestly significant difference (HSD) test for multiple comparisons. Statistical analyses were performed using Microsoft Excel 2016 and IBM SPSS Statistics 25. Statistical significance was indicated as follows: ***p < 0.001, **p < 0.01, *p < 0.05. Excitation-emission matrix data were resolved by parallel factor analysis (PARAFAC) in MATLAB R2024b, and general graphical visualization was performed in Origin 2026. Microbial community analyses were conducted in R 4.6.1 using the vegan and related packages. Principal coordinate analysis (PCoA) based on Bray-Curtis dissimilarities was used to visualize differences in bacterial community composition, and analysis of similarities (ANOSIM) was performed to test the statistical separation among groups. Spearman rank correlation analysis and Mantel tests were also performed in R4.6.1. *p* values from multiple comparisons were adjusted using the Benjamini-Hochberg false discovery rate procedure.

## 3 Results and discussion

### 3.1 Mineral characterization

**Figure 1:**
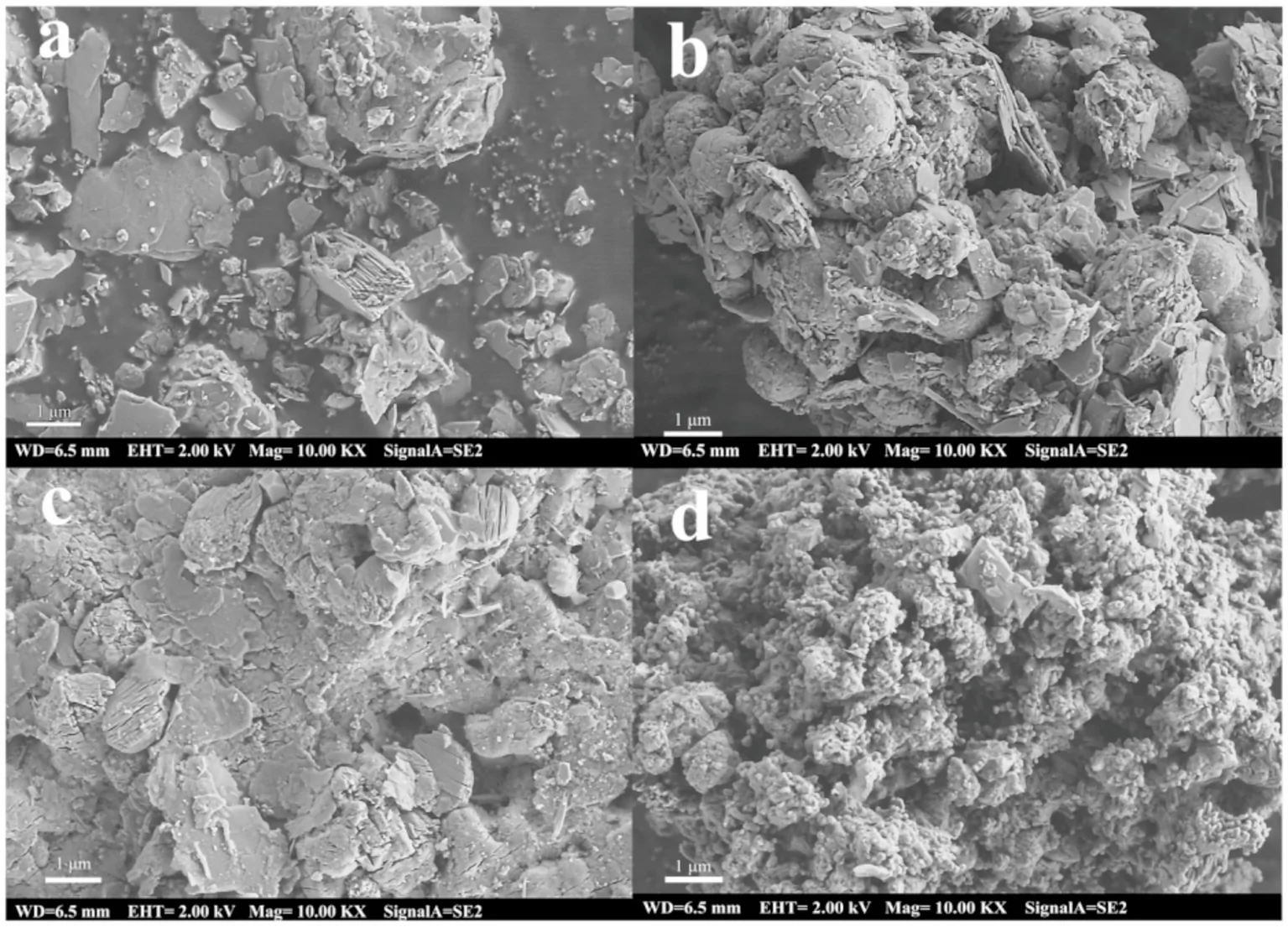
Different mineral scanning electron microscope images: **(a)** Shale, **(b)** mullite-based soil conditioner, **(c)** desulfurization gypsum, and **(d)** rare earth silicon titanium ore powder.

The four minerals exhibited distinct surface morphologies and pore structures (Figure 1). Shale was composed mainly of irregular lamellae that overlapped to form abundant slit-like voids. Mullite contained both plate-like particles and rounded aggregates, producing a relatively open interparticle structure. Desulfurization gypsum consisted of loosely stacked tabular crystals with comparatively smooth surfaces, whereas rare-earth silicon-titanium ore formed compact agglomerates with fewer visible open pores. The N₂ adsorption–desorption isotherms exhibited mesoporous characteristics with H3-type hysteresis loops (Supplementary Figure S1), indicating slit-shaped pores and interparticle voids formed by aggregated particles. BET analysis further demonstrated the structural differences among the minerals. Shale had the largest specific surface area (9.82 m^2^·g^−1^) and the smallest average pore diameter (12.68 nm), providing abundant surface adsorption sites. Mullite combined a comparable surface area (9.65 m^2^·g^−1^) with a high pore volume (0.0201 cm^3^·g^−1^) and an average pore diameter of 28.99 nm. Desulfurization gypsum exhibited the largest pore volume (0.0208 cm^3^·g^−1^) and average pore diameter (29.88 nm), although its surface area was lower (8.10 m^2^·g^−1^). Rare-earth silicon-titanium ore powder had the lowest surface area (3.53 m^2^·g^−1^) and pore volume (0.0044 cm^3^·g^−1^) (Supplementary Table S3). In addition to their contrasting pore structures, the amendments also differed in mineral composition and chemical characteristics (Supplementary Table S1), which may further influence mineral–organic interactions during composting. Surface area, pore volume and mineral surface chemistry jointly regulate the retention and transformation of organic matter at mineral interfaces (Tong et al., 2024; Xu and Tsang, 2024). Accordingly, the high density of surface sites in shale and the combined surface-area–pore-volume characteristics of mullite may have favored interactions with compost-derived organic components, whereas the less-developed pore structure of TO may have provided lower adsorption potential. Similar mineral-dependent differences in organic component stabilization have been reported during mineral-amended composting (Jiao et al., 2025).

### 3.2 Physicochemical properties of composting

**Figure 2:**
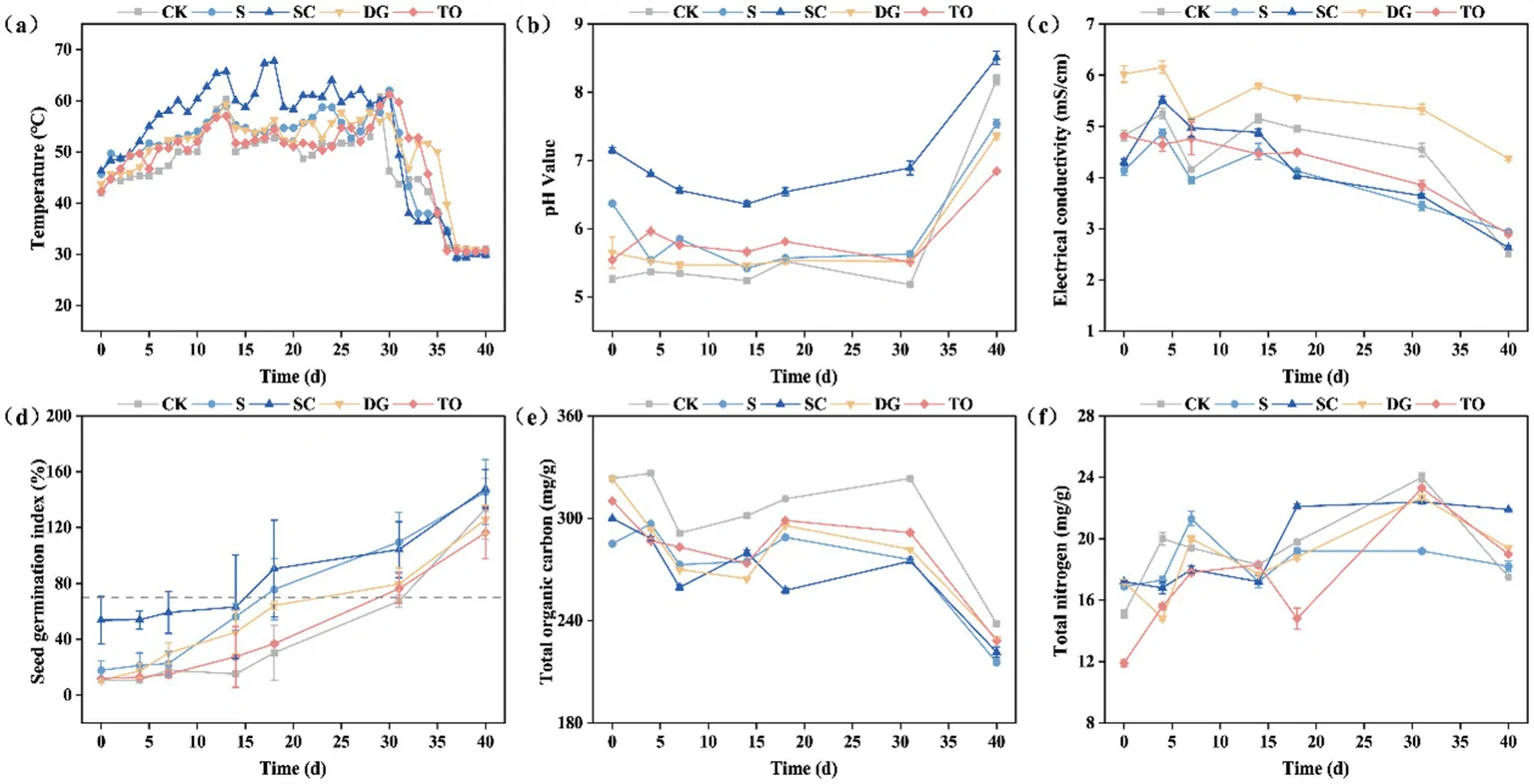
Changes in physicochemical properties during 40-day industrial-scale spent mushroom substrate composting under different mineral treatments: **(a)** Temperature, **(b)** pH value, **(c)** electric conductivity, **(d)** germination index, **(e)** total organic carbon, and **(f)** total nitrogen.

Mineral addition reshaped the thermal evolution of composting (Figure 2a; Noor et al., 2024). CK first exceeded 50 °C on day 8 and remained thermophilic for 13 consecutive days, whereas the mineral-amended composting reached 50 °C within 4–6 d and maintained thermophilic conditions for 27–28 d. SC exhibited the greatest thermophilic intensity, reaching 67.7 °C on day 18, while S, DG, and TO reached maxima of 62.0, 59.3, and 61.3 °C, respectively. Mineral addition therefore promoted pile heating, while SC most strongly enhanced thermophilic intensity. Studies have shown that mineral additives can improve pile aeration and promote thermophilic bacteria (Huo et al., 2023). Mineral-dependent pore structure, surface chemistry, and pile porosity may have influenced oxygen transfer, substrate accessibility, and microbial metabolism (Tong et al., 2024). Although shale had the largest surface area, DG and TO maintained thermophilic conditions for comparatively long periods despite their less-developed surface structures. The pronounced response of SC may be associated with its combined surface-area and pore-volume characteristics, which could provide favorable microsites for microbial colonization and substrate transformation (Barthod et al., 2018).

The pH of all treatments increased during composting (Figure 2b). SC maintained the highest pH throughout the experiment and reached 8.50 at maturation, followed by CK (8.18), S (7.54), DG (7.36) and TO (6.84). The increase in pH was mainly associated with the degradation of organic acids and ammonification of organic nitrogen during the active phase (Carreño et al., 2026; Hoang et al., 2022). EC generally declined toward maturation (Figure 2c), with final values of 2.51, 2.94, 2.63, 4.37 and 2.90 mS·cm^−1^ in CK, S, SC, DG and TO, respectively. DG maintained the highest EC throughout composting, which may be related to the dissolution of CaSO₄ in desulfurization gypsum increased the concentrations of soluble Ca^2+^ and SO₄^2−^ (Zhao et al., 2020). GI increased continuously in all treatments and exceeded 100% at day 40 (Figure 2d), indicating reduced phytotoxicity and advanced compost maturity (Kong et al., 2024). SC and S achieved the highest final GI values of 147.8 and 145.5%, respectively, compared with 133.5% in CK, 125.7% in DG and 116.4% in TO. TOC decreased by 24.4–29.0% during composting (Figure 2e), indicating extensive mineralization of organic matter. SC showed a rapid early-stage decline in TOC accompanied by higher temperatures, consistent with more rapid organic matter loss during the active phase. DG exhibited the greatest overall TOC loss (29.0%), indicating a greater apparent loss of organic carbon during composting. TN increased relative to the initial value in all treatments (Figure 2f), reaching 17.5 mg·g^−1^ in CK and 18.2–21.9 mg·g^−1^ in the mineral-amended treatments. The enrichment of TN was related to organic carbon loss and the progressive concentration of nitrogen-containing components, whereas mineral composition, pH and ion-exchange properties may also have influenced nitrogen transformation (He et al., 2024; Hoang et al., 2022). Overall, mullite achieved the most balanced improvement in thermophilic performance and compost maturity, followed by shale, whereas desulfurization gypsum was associated with greater TOC loss but also higher EC.

#### 3.2.1 Transformation of humus fractions

**Figure 3:**
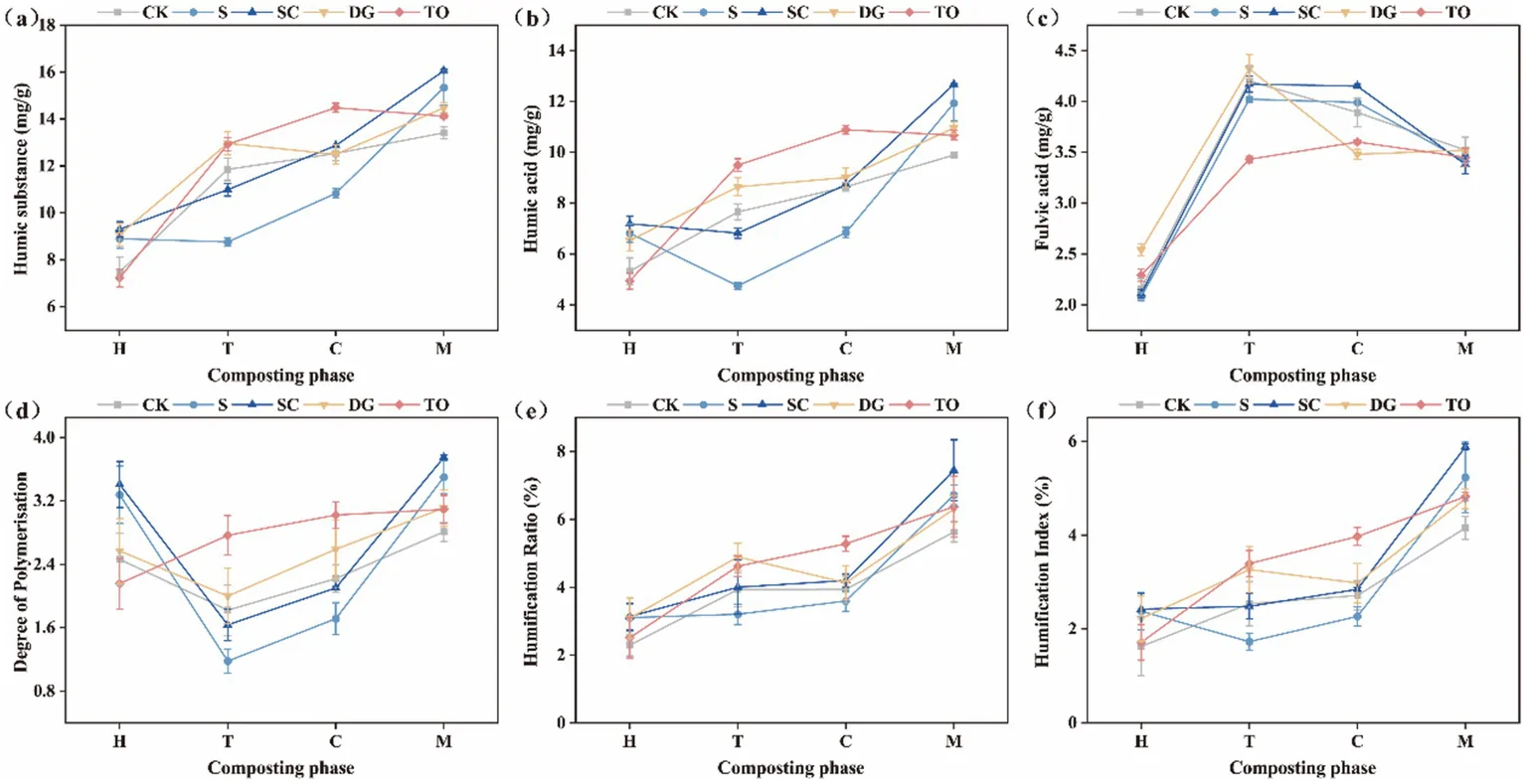
Changes in humus fractions and humification indices during composting: **(a)** Humic substance content, **(b)** humic acid content, **(c)** fulvic acid content, **(d)** degree of polymerization, **(e)** humification ratio, and **(f)** humification index. CK, control; S, shale powder; SC, mullite-based soil conditioner; DG, flue-gas desulfurization gypsum; and TO, rare-earth silico-titanate ore powder. H, T, C, and M represent the heating, thermophilic, cooling, and maturation phases sampled on days 0, 18, 31, and 40, respectively. Values are presented as mean ± SD of three analytical measurements (*n* = 3). Differences among treatments at the same composting phase were evaluated using one-way ANOVA followed by Tukey’s HSD test (*p* < 0.05).

Compost maturation involves not only the degradation of organic matter but also the transformation of organic components into more polymerized humic substances. Humic substances accumulated throughout composting, with clear differences among mineral-amended treatments (Figure 3a). At maturity, HS reached 15.34 and 16.05 mg g^−1^ in S and SC, respectively, representing increases of 14.4 and 19.7% relative to CK, both were significantly higher than CK (*p* < 0.05; Supplementary Table S4). Mineral surfaces may promote humification by adsorbing and concentrating humus precursors while providing reactive interfaces for subsequent biotic and abiotic transformations (Bui et al., 2023; X. Gao et al., 2024). The most HS accumulation in SC was consistent with its relatively balanced surface area-pore volume characteristics and stronger thermophilic response.

The increase in HS was mainly driven by HA formation (Figure 3b). During the thermophilic phase, HA contents decreased to 4.74 and 6.82 mg g^−1^ in S and SC, respectively, and were significantly lower than those in CK, DG, and TO (*p* < 0.05; Supplementary Table S4). The transient decrease in extractable HA under S and SC may reflect a redistribution of humic fractions during active composting, potentially arising from the combined effects of mineral-organic interactions and microbial transformation (Zhang et al., 2022). From the cooling to maturation phase, HA increased by 74.4% in S and 45.3% in SC, indicating sustained HA formation during the later stage of composting. Final HA contents in S and SC reached 11.93 and 12.67 mg·g^−1^, respectively, representing increases of 20.6 and 28.1% relative to CK. Both S and SC showed significantly higher final HA contents than CK, DG, and TO (*p* < 0.05), while no significant difference was observed between S and SC. In contrast, FA showed a transient increase followed by a decline in all treatments (Figure 3c). Its initial accumulation may reflect the release of low-molecular-weight humus precursors during organic matter decomposition, whereas the subsequent decline was consistent with microbial utilization and transformation into more polymerized humus fractions (Marge et al., 2022; Qi et al., 2021). At the end of composting, final FA contents varied only slightly among treatments, ranging from 3.38 to 3.52 mg·g^−1^, with no significant differences among treatments (*p* > 0.05). This indicates that the mineral-induced differences in HS accumulation were primarily associated with HA formation rather than FA retention.

The humification indices further confirmed the mineral-specific effects (Figures 3d–f). At the end of composting, SC exhibited the highest HI, HR, and DP values of 5.88, 7.45%, and 3.75, respectively, representing increases of 41.5, 32.2, and 33.5% relative to CK. SC showed significantly higher HI and DP than CK, DG, and TO, and significantly higher HR than CK (*p* < 0.05). The elevated DP was consistent with a greater predominance of HA relative to FA and a higher degree of humification, whereas the increases in HI and HR reflected greater HS stabilization and compost maturity (Wei et al., 2022). S showed the second-highest HI and DP values of 5.23% and 3.50, respectively, further demonstrating its positive effect on humification. In contrast, DG and TO showed intermediate humification responses at maturation despite their distinct temporal patterns of HA accumulation. Mineral surfaces can influence humification by regulating both microbial transformation and abiotic reactions of humus precursors (Pan et al., 2021). The abundant surface sites of shale may favor precursor retention, whereas the combined surface area and pore volume of mullite may enhance precursor adsorption, microbial colonization, and condensation reactions. The superior humification performance of SC despite its specific surface area being comparable to that of S is consistent with evidence that mineral effects depend on pore accessibility and surface reactivity rather than surface area alone (Song et al., 2023; Zheng et al., 2022). Overall, the numerical humification response at maturation generally followed the order SC > S > DG/TO > CK, with SC showing the highest overall levels of HS polymerization and humification and S exhibiting comparable enhancement in several key humification indices.

### 3.3 Evolution of DOM fluorescence components

**Figure 4:**
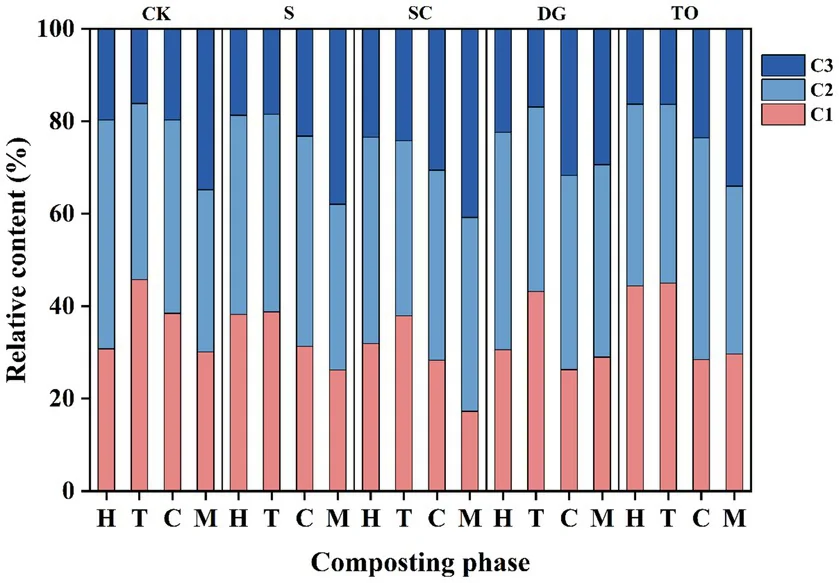
Mineral-specific changes in DOM fluorescence components during composting. C1 represents a fulvic-like component, whereas C2 and C3 represent humic-like components. H, T, C, and M correspond to days 0, 18, 31, and 40, respectively.

PARAFAC analysis resolved three fluorescent components, including one fulvic-like component (C1) and two humic-like components (C2 and C3) (Figure 4; Supplementary Figures S2, S3). During composting, the fluorescent DOM pool was progressively redistributed from fulvic-like toward more humic-like fluorescence components. This pattern is generally associated with the depletion of readily degradable organic matter, transformation of microbial metabolites, and gradual accumulation of humic-like substances (Xiong, 2022). In the CK, DG, and TO treatments, the proportion of C1 initially increased and subsequently declined, whereas C3 increased markedly during maturation. The transient accumulation of C1 may reflect the release of soluble intermediates during the degradation of macromolecular organic matter, whereas the subsequent increase in C3 was consistent with a relative shift toward more humic-like fluorescent DOM. Similar shifts in fluorescent components have been attributed to the combined effects of labile substrate degradation and the accumulation of humic substance during composting (Xin et al., 2023).

Mineral amendments markedly altered the relative distribution of DOM fluorescence components at maturation. The S treatment showed a decrease in the proportion of C1 from 38.21 to 26.20%, accompanied by an increase in C3 from 18.67 to 37.97%. A stronger shift was observed in SC, where C1 decreased from 31.93 to 17.26% and C3 increased from 23.41 to 40.81%. Consequently, SC exhibited the lowest C1 proportion and the highest C3 proportion at maturation, reflecting the most pronounced redistribution of fluorescent DOM toward the C3 humic-like component. In DG, C2 accounted for 41.67% of fluorescent DOM at maturity, whereas C3 represented only 29.34%, indicating that desulfurization gypsum preferentially favored the relative accumulation of the C2 humic-like component but had a comparatively limited effect on C3 enrichment. At maturity, the proportions of C1, C2, and C3 in the TO treatment were 29.69, 36.31, and 34.00%, respectively, closely resembling those in CK (30.11, 35.09, and 34.80%, respectively). This similarity indicates that rare-earth silicon-titanium ore powder had only a limited effect on the final distribution of fluorescent DOM components. These contrasting patterns indicated that the mineral amendments exerted component-specific effects on DOM evolution.

The increase in the C3 proportion under S and SC treatments was consistent with their higher HA contents, HI, and DP values, suggesting that the shift toward humic-like fluorescence occurred in parallel with changes in independently measured humification-related parameters. Comparable enrichment of humic-like fluorescence has been reported during full-scale composting, although component distributions vary with feedstock and extraction fraction (Rueda et al., 2024). This correspondence may reflect both the continuous production of humic precursors and the selective preservation of aromatic organic components by the minerals. Surface adsorption sites and reactive functional groups can retain aromatic, lignin derived, and oxygen-rich organic molecules through surface complexation, cation bridging, and pore entrapment, thereby reducing their subsequent degradation and mineralization and favoring the accumulation of humic-like components (Li et al., 2022; Wang et al., 2024). Mineral interfaces may also facilitate the adsorption, concentration, and structural rearrangement of humic precursors (Shen et al., 2025). Correlation analysis further showed that specific surface area and micropore volume were positively associated with HA, HI, and DOM C3 (Supplementary Figure S4), supporting the close relationship between mineral structural properties and compost humification. Taken together, the humus fractions and PARAFAC results indicated that both mullite and shale were associated with the development of high humification characteristics in composting DOM, with mullite exerting a more pronounced effect.

### 3.4 Mineral amendments reshaped bacterial community succession

**Figure 5:**
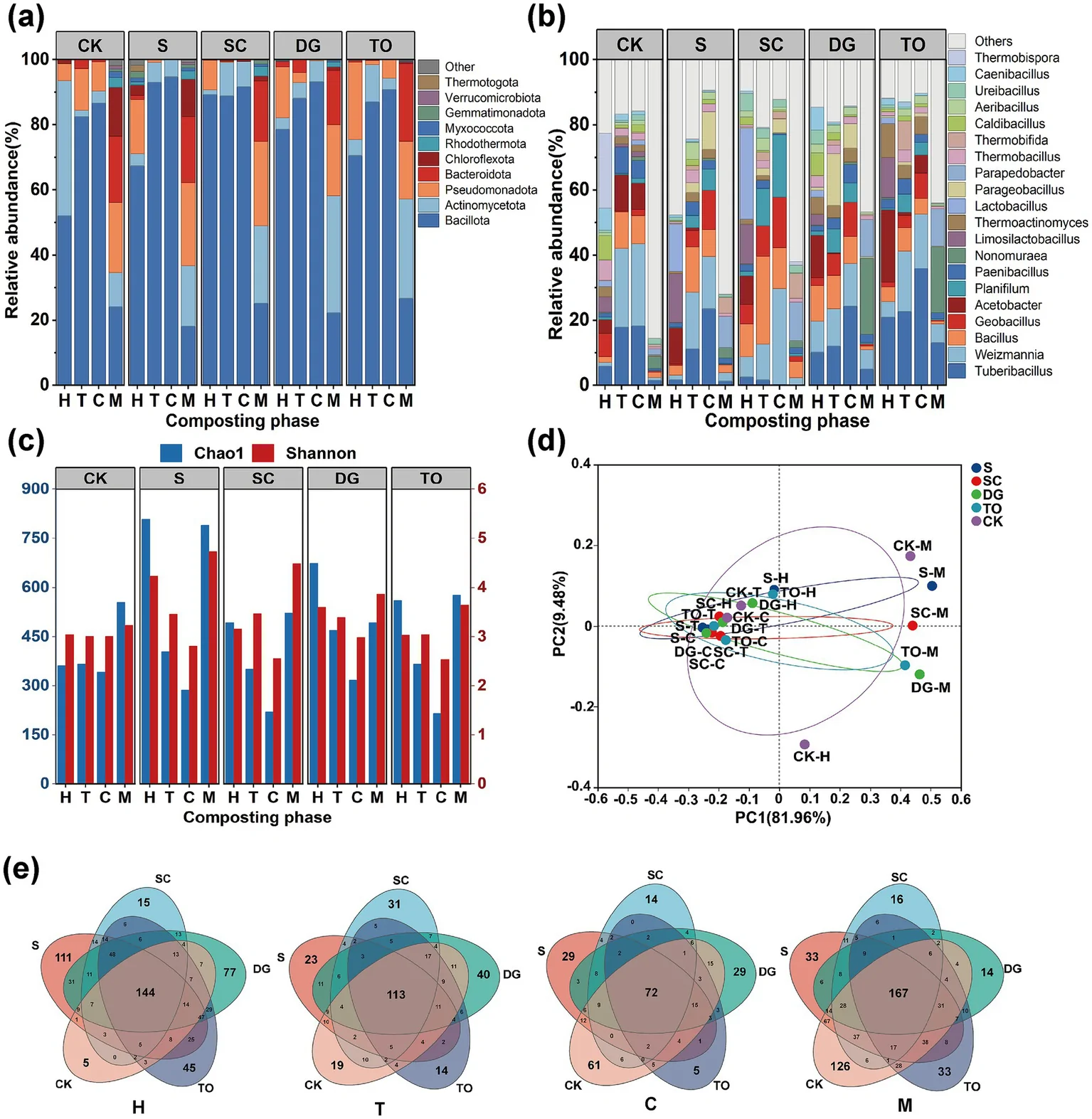
Changes in bacterial community composition and diversity during composting. **(a)** Relative abundance of the 10 most abundant phyla, **(b)** relative abundance of the 20 most abundant genera, **(c)** Chao1 richness and Shannon diversity indices, **(d)** principal coordinate analysis (PCoA) based on Bray-Curtis dissimilarities, **(e)** Venn diagrams, shared and treatment-specific bacterial OTUs at each phase. H, T, C, and M represent the heating, thermophilic, cooling, and maturation phases, respectively.

Bacterial community composition underwent pronounced temporal succession, with treatment-associated differences emerging at specific composting stages (Figure 5). At the phylum level (Figure 5a), Bacillota dominated the heating, thermophilic, and cooling phases, accounting for 51.97–94.74% of the bacterial community. This predominance indicated that thermotolerant and spore-forming bacteria were major participants in organic matter decomposition during the early and intermediate stages of composting (López-González et al., 2015; Meng et al., 2024). At maturation, the relative abundance of Bacillota declined to 18.11–26.71%, accompanied by increases in Actinomycetota, Pseudomonadota, and Bacteroidota. This succession reflected a transition from bacterial groups adapted to rapid substrate decomposition under thermophilic conditions toward those associated with the transformation of recalcitrant organic matter and late-stage compost maturation. Actinomycetota accounted for 35.80 and 30.47% of the bacterial communities in the DG and TO at maturation, respectively, compared with only 10.31% in CK. In contrast, Chloroflexota remained relatively abundant in CK and S (15.06 and 11.56%, respectively), but accounted for only 0.56–1.54% in SC, DG, and TO. These shifts indicate that the common temperature-driven succession of compost bacteria was further differentiated by mineral type. Similar phase-dependent selection of Bacillota and Actinomycetota has been reported during composting, while porous mineral additives can selectively alter thermophilic and humification-associated taxa (Ma et al., 2023; Zhu et al., 2021).

Genus-level composition further revealed phase-dependent responses to the different mineral amendments (Figure 5b). During the heating phase, *Lactobacillus* and *Limosilactobacillus* together accounted for 29.58 and 40.02% of the bacterial communities in S and SC, respectively, whereas *Acetobacter* represented 12.99 and 22.29% in DG and TO. These differences suggest that the mineral amendments altered the early-stage composting environment, thereby favoring bacterial taxa with distinct substrate-utilization preferences (Ma et al., 2023). During the thermophilic and cooling phases, several thermophilic or thermotolerant genera, including *Tuberibacillus*, *Weizmannia*, *Bacillus*, *Geobacillus*, *Planifilum*, and *Parageobacillus*, increased in relative abundance. Some members of these taxa have been associated with extracellular enzyme production and the degradation of proteins, cellulose, and other complex substrates (Bidart et al., 2023; Wang et al., 2020; Zhu et al., 2020). At maturation, the relative abundance of *Parapedobacter* increased to 9.62–11.85% across the mineral-amended treatments. *Nonomuraea* was particularly enriched in DG and TO, reaching 23.46 and 20.18%, respectively, compared with 1.90–3.66% in CK, S, and SC. As *Nonomuraea* belongs to Actinomycetota, its enrichment was consistent with the phylum-level increase in Actinomycetota, suggesting a stronger association of DG and TO with Actinomycetota during maturation. Overall, mineral amendment influenced not only the phase-dependent replacement of dominant genera but also taxon-specific differences in bacterial composition during maturation.

The Chao1 and Shannon indices generally decreased during the thermophilic-to-cooling transition and then recovered at maturation (Figure 5c), indicating strong high-temperature filtering followed by the re-establishment of bacterial richness and diversity as temperature decreased and substrate heterogeneity increased (Sun et al., 2021). S exhibited the highest initial richness and diversity (Chao1 = 807.91; Shannon = 4.23), whereas CK and S showed the highest values during maturation phase (Chao1 = 914.24 and 790.13; Shannon = 4.90 and 4.73, respectively). PCoA explained 91.44% of the total Bray–Curtis variation on the first two axes and revealed a pronounced temporal shift in bacterial community composition, with most maturation-stage samples separated from earlier stages (Figure 5d). However, ANOSIM detected no significant overall difference among mineral treatments (*R* = −0.137, *p* = 0.98). Stage-matched comparisons further showed that the DG and TO communities were most distant from CK during maturation phase (Bray–Curtis dissimilarity = 0.699 and 0.725), whereas SC displayed the largest average displacement from CK across all phases (0.618; Supplementary Figure S4). Thus, the influence of SC extended across multiple composting phases, whereas the effects of DG and TO became more pronounced during maturation, highlighting the phase-dependent effects of mineral amendments on bacterial community structure. Venn diagram analysis showed that the common bacterial pool contracted toward the cooling phase and expanded again at maturation, while each mineral-amended treatment retained phase-specific taxa (Figure 5e). Bacterial co-occurrence network analysis and associated topological indices further revealed that different mineral amendments reshaped potential bacterial association patterns (Supplementary Figure S5; Supplementary Table S4) (Siles et al., 2021). Compared with CK (167 nodes and 1,431 edges, 0.674), mineral-amended networks contained fewer edges (520–1,152) but higher modularity (0.748–0.903). Negative associations also increased from 11.88% in CK to 25.96–38.65% following mineral addition; SC had the highest modularity (0.903), whereas DG had the greatest negative-edge proportion (38.65%). Comparable mineral-dependent community restructuring has been reported during vermiculite-amended sludge-mushroom residue co-composting (Yu et al., 2024), suggesting that mineral type may modify network organization as well as taxonomic succession. The combination of reduced connectivity and enhanced compartmentalization indicates a shift toward a more compartmentalized co-occurrence network structure rather than uniformly strengthening bacterial associations. The increased proportion of negative associations may reflect greater ecological differentiation among bacterial taxa (Srinivasan et al., 2024). Given the close association between microbial network architecture and organic matter transformation during composting (Meng et al., 2024), such mineral-specific restructuring may partly underlie the observed differences in substrate turnover and humification among treatments.

### 3.5 Core bacterial succession associated with humification

**Figure 6:**
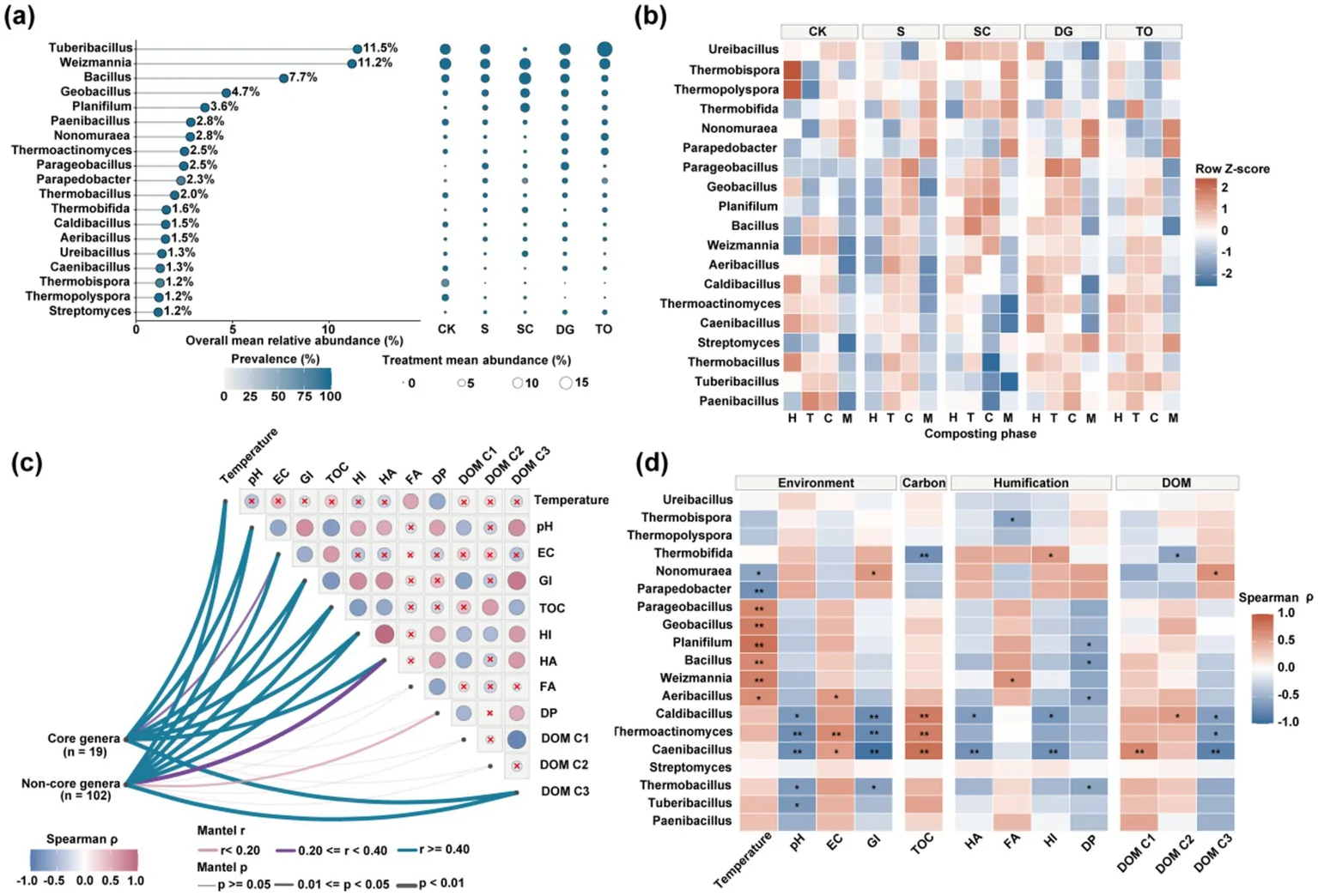
Distribution of core bacterial genera in mineral-amended composts and associations with physicochemical properties, humification indices, and DOM components: **(a)** Overall mean relative abundance, prevalence, and treatment-level distribution of 19 core genera. Bubble size and color denote treatment-level mean abundance and prevalence, respectively; core genera were defined by prevalence ≥ 80% across the 20 samples and mean relative abundance ≥ 1%. **(b)** Row Z-score heatmap across CK, S, SC, DG, and TO at H, T, C, and M (days 0, 18, 31, and 40). **(c)** Mantel associations of core and non-core genus assemblages with composting, humification, and DOM variables. Link colors denote Mantel *r* classes (*r* < 0.20, 0.20 ≤ *r* < 0.40, and *r* ≥ 0.40), and link widths denote FDR-adjusted *p* classes (*p* ≥ 0.05, 0.01 ≤ *p* < 0.05, and *p* < 0.01). The upper triangle shows Spearman correlations among variables; red crosses indicate *p* ≥ 0.05. **(d)** Spearman correlations between individual core genera and selected variables; red and blue indicate positive and negative coefficients, respectively, with *p < 0.05 and **p < 0.01.

To further characterize the persistent bacterial taxa involved in mineral-induced community shifts, 19 global core bacterial genera were identified across all composting stages and treatments, with 16 consistently detected in all samples (Figure 6a). *Tuberibacillus*, *Weizmannia*, and *Bacillus* were the most abundant core genera, with overall mean relative abundances of 11.5, 11.2, and 7.7%, respectively. Although these genera represented a shared bacterial foundation, their relative contributions differed considerably among the four minerals. SC was characterized by relatively high abundances of *Bacillus*, *Weizmannia*, *Planifilum*, *Geobacillus*. Both S and DG maintained relatively high abundances of *Tuberibacillus*, *Weizmannia*, and *Bacillus*, whereas DG additionally showed relatively high abundances of *Parageobacillus* and *Nonomuraea*. TO was characterized by a pronounced dominance of *Tuberibacillus*. Zhan et al. (2023) reported the persistence of core microbial communities across composting stages, while their relative abundances and ecological associations were selectively shaped by feedstock characteristics and environmental conditions. Thus, the mineral-specific response was primarily reflected in the redistribution of the shared core microbiota rather than the establishment of entirely distinct core communities.

The heatmap revealed distinct successional patterns among the core genera (Figure 6b). SC showed relatively high abundances of *Bacillus*, *Geobacillus*, *Planifilum*, and *Thermobifida* during the thermophilic and cooling stages. These genera have strong potential for extracellular enzyme production and lignocellulose degradation, which may enhance the release of humic precursors and thereby promote compost humification (Qin et al., 2025; Zhou et al., 2023). This pattern was consistent with the higher HA, HI, DP, and DOM C3 values observed in SC. S maintained a more balanced core bacterial community and showed a comparatively moderate humification response. In contrast, DG and TO mainly enriched *Nonomuraea* and *Parapedobacter* during maturation. *Nonomuraea* has been implicated in hemicellulose and lignocellulose degradation after the thermophilic phase, whereas *Parapedobacter* has been associated with polysaccharide hydrolysis and compost humification (Qin et al., 2025; Zhang et al., 2024). This may facilitate the continued transformation of residual recalcitrant organic matter during maturation, thereby promoting compost humification. These treatment-specific successional patterns were consistent with the contrasting humification responses observed among the mineral amendments.

Mantel analysis further revealed significant associations between core bacterial community composition, composting conditions, and humification-related properties (Figure 6c). core-community composition was significantly associated with temperature, pH, EC, GI, TOC, HI, HA, and DOM C3 (Mantel *r* = 0.323–0.728, FDR-adjusted *p* < 0.05). Non-core genera exhibited a broadly similar pattern and were additionally associated with DP. These results indicate that humification was coupled with coordinated changes in both core and non-core bacterial community fractions. Comparable links among temperature, organic-carbon transformation, bacterial succession, and HA formation have been reported in other composting systems (Gao Y. et al., 2024; Meng et al., 2022). Genus-level Spearman analysis further revealed distinct associations between core bacterial genera and composting conditions and humification-related indicators across mineral-amended treatments (Figure 6d). *Nonomuraea* was positively correlated with GI and DOM C3 but negatively correlated with temperature, consistent with its enrichment at maturation and in DG and TO. *Thermobifida*, which was relatively abundant in SC, was positively associated with HI but negatively associated with TOC and DOM C2. Previous studies have also identified *Thermobifida* as a widespread core composting genus associated with HA accumulation (Li et al., 2023; Zhan et al., 2023). Conversely, *Caenibacillus* and *Caldibacillus* were positively associated with TOC or less-humified DOM components but negatively associated with HA, HI, GI, or DOM C3. This pattern may reflect the preferential occurrence of these genera under conditions prevailing during the heating and thermophilic phases. As substrate and environmental conditions changed, their abundances declined, and they were replaced by maturation-associated taxa. Collectively, these patterns suggest that mineral-specific shifts in core-genera abundance were closely aligned with differences in organic-carbon transformation, humus accumulation, and DOM composition. Overall, mineral amendments reshaped the succession of core bacterial genera. SC showed greater representation of genera associated with the thermophilic and cooling stages together with higher humification indices, S maintained a comparatively balanced assemblage, whereas DG and TO showed greater representation of maturation-associated genera.

### 3.6 Proposed coupled abiotic and biotic pathways underlying mineral-specific humification

**Figure 7:**
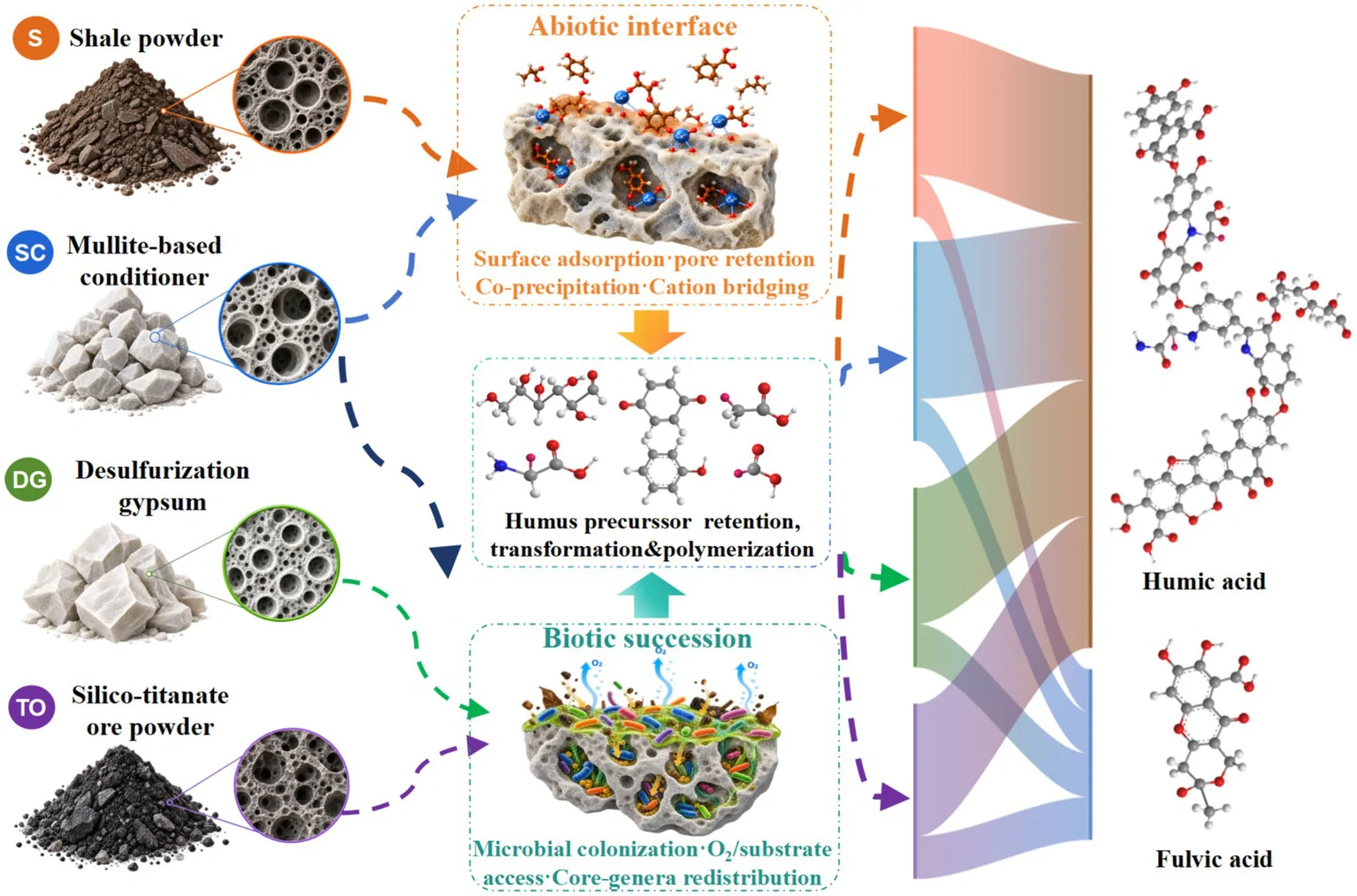
Proposed coupled abiotic-biotic framework for mineral-specific humification. Proposed abiotic pathways include surface adsorption, co-precipitation, cation bridging, and pore-scale precursor retention; the proposed biotic pathway involves microbial colonization, O_2_/substrate access, and core-genera redistribution. Dashed arrows denote mechanistic hypotheses inferred from the combined physicochemical, humification, DOM, and bacterial evidence and do not represent directly measured causal fluxes.

The integrated evidence suggests that mineral-specific humification may arise from the coupling of abiotic mineral-organic interactions and stage-dependent microbial succession (Figure 7). At the abiotic interface, adsorption, surface co-precipitation, cation bridging, and pore-scale confinement can promote the retention and local enrichment of humus precursors, thereby increasing their opportunity for subsequent condensation and polymerization while limiting further mineralization (Gao X. et al., 2024; Pan et al., 2021; Tong et al., 2024). The contrast between S and SC is particularly informative. Although S had the highest specific surface area, SC combined a comparable surface area with approximately twice the pore volume and a substantially larger mean pore diameter. This structural advantage coincided with the greatest increases in HA, HI, and the humic-like C3 component, indicating that surface area alone did not determine humification performance. Beyond pore architecture, differences in mineral composition and chemical characteristics may also contribute to these contrasting responses by altering interfacial reactivity and the availability of soluble ionic species. In contrast, CaSO₄-rich DG preferentially enhanced C2, suggesting a component selective effect on DOM transformation, whereas the compact, poorly developed pore structure of TO was associated with only minor changes in final DOM composition.

The microbial patterns further support this interpretation. SC showed greater representation of substrate-transforming core genera during the thermophilic and cooling phases, whereas DG and TO were more strongly associated with maturation-related Actinomycetota. For S, its high specific surface area may have favored interfacial precursor retention, but its lower pore volume and smaller mean pore diameter than SC likely provided less favorable microsites for microbial colonization and substrate exchange. In contrast, SC combined developed mesoporosity with reactive mineral interfaces, potentially coupling microbial transformation with the retention of intermediate products and thereby favoring their subsequent condensation and polymerization into humic substance. DG may have followed a more ion-mediated pathway associated with its CaSO₄-rich composition, as dissolution of Ca-bearing phases could increase the availability of soluble ionic species and facilitate ion-mediated mineral–organic interactions, consistent with its C2-selective DOM response. However, the compact structure and low surface area of TO may have constrained both precursor retention and microbial transformation. Collectively, these patterns link mineral properties, DOM evolution, and bacterial succession to the divergent humification responses among treatments.

## 4 Conclusion

Mineral origin and types determined distinct patterns of organic matter transformation, humification, and bacterial succession during industrial-scale spent mushroom substrate composting. Overall, Mineral amendments accelerated pile heating, prolonged the thermophilic phase, promoted humus formation, and enhanced compost maturation. The mullite-based soil conditioner showed the strongest integrated performance, increasing HA content by 28.1% and humification indices by 32.2–41.5% relative to CK, while promoting the conversion of DOM into more complex humic-like components. Mineral-specific redistribution of the shared core bacterial community paralleled the contrasting humification patterns. SC favored heat-adapted core genera associated with organic matter transformation and humification during the thermophilic and cooling phases, whereas shale maintained a comparatively balanced assemblage and DG and TO selected maturation-associated Actinomycetota such as *Nonomuraea*. Overall, the mullite-based conditioner was the most effective amendment for improving SMS compost maturity and humification. These findings provide a practical basis for developing low-cost mineral-amended composting strategies.

## Data Availability

The data analyzed in this study is available in the NCBI BioProject repository (https://www.ncbi.nlm.nih.gov/bioproject) under accession number PRJNA1502519.

## References

[ref1] Babu S. Rathore S. S. Singh R. Kumar S. Singh V. K. Yadav S. . (2022). Exploring agricultural waste biomass for energy, food and feed production and pollution mitigation: a review. Bioresour. Technol. 360:127566. doi: 10.1016/j.biortech.2022.127566 35788385

[ref2] Barthod J. Rumpel C. Dignac M. F. (2018). Composting with additives to improve organic amendments. A review. Agron. Sustain. Dev. 38:17. doi: 10.1007/s13593-018-0491-9

[ref3] Bidart G. N. Gharabli H. Welner D. H. (2023). Functional characterization of the phosphotransferase system in Parageobacillus thermoglucosidasius. Sci. Rep. 13:7131. doi: 10.1038/s41598-023-33918-1, 37130962 PMC10154347

[ref4] Brunchi C.-E. Morariu S. (2024). Laponite®—from dispersion to gel—structure, properties, and applications. Molecules 29:2823. doi: 10.3390/molecules29122823, 38930887 PMC11206873

[ref5] Bui V. K. H. Truong H. B. Hong S. Li X. Hur J. (2023). Biotic and abiotic catalysts for enhanced humification in composting: a comprehensive review. J. Clean. Prod. 402:136832. doi: 10.1016/j.jclepro.2023.136832

[ref6] Cao Y. Yang H. Liu Y. Kong F. Zhu Y. Chen Y. . (2025). Attapulgite-modified organic compost effectively reduces soil nutrient loss and enhances microbial interactions. J. Environ. Sci. 162, 405–416. doi: 10.1016/j.jes.2025.04.065, 41765539

[ref7] Carreño G. Marambio R. Valenzuela U. A. Aponte H. Floody M. C. Soto R. I. C. . (2026). Effect of biochar as an additive in co-composting: impacts on physicochemical properties, enzyme activity, and substrate quality. Agronomy 16:1268. doi: 10.3390/agronomy16131268

[ref8] Chang Y. Zhou K. Yang T. Zhao X. Li R. Li J. . (2023). *Bacillus licheniformis* inoculation promoted humification process for kitchen waste composting: organic components transformation and bacterial metabolic mechanism. Environ. Res. 237:117016. doi: 10.1016/j.envres.2023.117016, 37657603

[ref9] Chen L. Chen Y. Li Y. Liu Y. Jiang H. Li H. . (2023). Improving the humification by additives during composting: a review. Waste Manag. 158, 93–106. doi: 10.1016/j.wasman.2022.12.040, 36641825

[ref10] Gao Y. Liu S. Wang N. Wang Y. (2024). Humic acid biosynthesis and bacterial community evolution during aerobic composting of rice straw. Appl. Microbiol. Biotechnol. 108:177. doi: 10.1007/s00253-023-12994-3, 38277012 PMC10817993

[ref11] Gao X. Zhang J. Liu G. Kong Y. Li Y. Li G. . (2024). Enhancing the transformation of carbon and nitrogen organics to humus in composting: biotic and abiotic synergy mediated by mineral material. Bioresour. Technol. 393:130126. doi: 10.1016/j.biortech.2023.130126, 38036150

[ref12] He W. Rong S. Wang J. Zhao Y. Liang Y. Huang J. . (2024). Different crystalline manganese dioxide and biochar co-conditioning aerobic composting: reduced ammonia volatilization and improved organic fertilizer quality. J. Hazard. Mater. 465:133127. doi: 10.1016/j.jhazmat.2023.133127, 38056255

[ref13] Hoang H. G. Thuy B. T. P. Lin C. Vo D. V. N. Tran H. T. Bahari M. B. . (2022). The nitrogen cycle and mitigation strategies for nitrogen loss during organic waste composting: a review. Chemosphere 300:134514. doi: 10.1016/j.chemosphere.2022.134514, 35398076

[ref14] Huang D. Gao L. Cheng M. Yan M. Zhang G. Chen S. . (2022). Carbon and N conservation during composting: a review. Sci. Total Environ. 840:156355. doi: 10.1016/j.scitotenv.2022.156355, 35654189

[ref15] Huo X. Yan Z. Chen M. Zhou J. Zheng C. (2023). Potassium-rich mining waste addition can shorten the composting period by increasing the abundance of thermophilic bacteria during high-temperature periods. Sci. Rep. 13:6027. doi: 10.1038/s41598-023-31689-3 PMC10101976 37055422

[ref16] Jiao Z. Li R. Zhang K. Zhang Y. Guo Y. Chang S. . (2025). From carbon sequestration perspective: adsorption of minerals enhances the stabilization of organic fractions in composting. Environ. Technol. Innov. 37:104023. doi: 10.1016/j.eti.2025.104023

[ref17] Khan R. Gao J. Abdellah Y. A. Y. Liu D. Yu F. (2026). Rethinking spent mushroom substrate: from lignocellulosic waste to soil–microbiome bioresource for circular agriculture. Bioresour. Technol. 461:135448. doi: 10.1016/j.biortech.2026.135448, 42476384

[ref18] Kong Y. Zhang J. Zhang X. Gao X. Yin J. Wang G. . (2024). Applicability and limitation of compost maturity evaluation indicators: a review. Chem. Eng. J. 489:151386. doi: 10.1016/j.cej.2024.151386

[ref19] Li Y. Gong X. Sun Y. Shu Y. Niu D. Ye H. (2022). High molecular weight fractions of dissolved organic matter (DOM) determined the adsorption and electron transfer capacity of DOM on iron minerals. Chem. Geol. 604:120907. doi: 10.1016/j.chemgeo.2022.120907

[ref20] Li Y. Li J. Chang Y. Li R. Zhou K. Zhan Y. . (2023). Comparing bacterial dynamics for the conversion of organics and humus components during manure composting from different sources. Front. Microbiol. 14:1281633. doi: 10.3389/fmicb.2023.1281633, 37840749 PMC10568323

[ref21] López-González J. Suárez-Estrella F. Vargas-García M. López M. Jurado M. Moreno J. (2015). Dynamics of bacterial microbiota during lignocellulosic waste composting: studies upon its structure, functionality and biodiversity. Bioresour. Technol. 175, 406–416. doi: 10.1016/j.biortech.2014.10.123, 25459849

[ref22] Ma S. Shen Y. Ding J. Cheng H. Zhou H. Ge M. . (2023). Effects of biochar and volcanic rock addition on humification and microbial community during aerobic composting of cow manure. Bioresour. Technol. 391:129973. doi: 10.1016/j.biortech.2023.129973, 37931759

[ref23] Ma X. Yan S. Wang M. (2025). Spent mushroom substrate: a review on present and future of green applications. J. Environ. Manag. 373:123970. doi: 10.1016/j.jenvman.2024.123970, 39754812

[ref24] Marge L. Maris K. Oskars P. Merrit S. Anu K. Mait K. (2022). Properties of humic substances in composts comprised of different organic source material. Agriculture 12:1797. doi: 10.3390/agriculture12111797

[ref25] Meng X. Liang X. Wang P. Ren L. (2024). Effect of thermophilic bacterial complex agents on synergistic humification of carbon and nitrogen during lignocellulose-rich kitchen waste composting. J. Environ. Manag. 370:122799. doi: 10.1016/j.jenvman.2024.122799, 39393336

[ref26] Meng L. Xu C. Wu F. (2022). Microbial co-occurrence networks driven by low-abundance microbial taxa during composting dominate lignocellulose degradation. Sci. Total Environ. 845:157197. doi: 10.1016/j.scitotenv.2022.157197 35839876

[ref27] Muscarella S. M. Badalucco L. Cano B. Laudicina V. A. Mannina G. (2021). Ammonium adsorption, desorption and recovery by acid and alkaline treated zeolite. Bioresour. Technol. 341:125812. doi: 10.1016/j.biortech.2021.125812, 34455254

[ref28] Noor R. S. Shah A. N. Tahir M. B. Umair M. Nawaz M. Ali A. . (2024). Recent trends and advances in additive-mediated composting technology for agricultural waste resources: a comprehensive review. ACS Omega 9, 8632–8653. doi: 10.1021/acsomega.3c06516, 38434807 PMC10905604

[ref29] Pan C. Zhao Y. Zhao L. Wu J. Zhang X. Xie X. . (2021). Modified montmorillonite and illite adjusted the preference of biotic and abiotic pathways of humus formation during chicken manure composting. Bioresour. Technol. 319:124121. doi: 10.1016/j.biortech.2020.124121, 32957045

[ref30] Qi H. Zhai W. Du Y. Zhao Y. Wei Z. Wu J. (2021). Core bacterial community driven the conversion of fulvic acid components during composting with adding manganese dioxide. Bioresour. Technol. 337:125495. doi: 10.1016/j.biortech.2021.125495, 34320772 34320772

[ref31] Qin X. Bao R. Huang W. Li Q. (2025). Facilitating the enzymatic hydrolysis of polysaccharides by carbohydrate active enzymes and enhanced humification process with microbial consortium revealed by metagenomics analysis during cow manure-straw composting. J. Environ. Chem. Eng. 13:115428. doi: 10.1016/j.jece.2025.115428

[ref32] Rangappa H. S. Herath I. Lin C. Ch S. (2024). Industrial waste-based adsorbents as a new trend for removal of water-borne emerging contaminants. Environ. Pollut. 343:123140. doi: 10.1016/j.envpol.2023.123140, 38103712

[ref33] Rueda M. P. Domínguez-Vidal A. Llorent-Martínez E. J. Aranda V. Ayora-Cañada M. J. (2024). Monitoring organic matter transformation of olive oil production residues in a full-scale composting plant by fluorescence spectroscopy. Environ. Technol. Innov. 35:103695. doi: 10.1016/j.eti.2024.103695

[ref34] Shen B. Zhang X. Tao W. Wang X. Wei Z. Song C. . (2025). Elucidating the clay-mediated fixation mechanism of humic acids in composting systems using LC-MS and spectroscopic techniques. Environ. Res. 283:122106. doi: 10.1016/j.envres.2025.122106, 40505951

[ref35] Siles J. A. García-Sánchez M. Gómez-Brandón M. (2021). Studying microbial communities through co-occurrence network analyses during processes of waste treatment and in organically amended soils: a review. Microorganisms 9:1165. doi: 10.3390/microorganisms9061165, 34071426 PMC8227910

[ref36] Song C. Gao Y. Sun Q. Zhao Y. Qi H. Chen Z. . (2023). Insight into the pathways of biochar/smectite-induced humification during chicken manure composting. Sci. Total Environ. 905:167298. doi: 10.1016/j.scitotenv.2023.167298, 37742972

[ref37] Srinivasan S. Jnana A. Murali T. S. (2024). Modeling microbial community networks: methods and tools for studying microbial interactions. Microb. Ecol. 87:56. doi: 10.1007/s00248-024-02370-7, 38587642 PMC11001700

[ref38] Sun L. Long M. Li J. Wu R. Ma L. Tang D. . (2021). Different effects of thermophilic microbiological inoculation with and without biochar on physicochemical characteristics and bacterial communities in pig manure composting. Front. Microbiol. 12:746718. doi: 10.3389/fmicb.2021.746718, 34899633 PMC8660119

[ref39] Tong Y. Xiang H. Jiang J. Chen W. (2024). Interfacial interactions between minerals and organic matter: mechanisms and characterizations. Chemosphere 359:142383. doi: 10.1016/j.chemosphere.2024.142383, 38768785

[ref40] Wang S. Liu T. Zhu E. He C. Shi Q. Feng X. (2024). Potential retention of dissolved organic matter by soil minerals during wetland water-table fluctuations. Water Res. 254:121412. doi: 10.1016/j.watres.2024.121412, 38457944

[ref41] Wang M. Miao J. Wang X. Li T. Zhu H. Liu D. . (2020). Genomic and transcriptome analyses of a thermophilic bacterium *Geobacillus stearothermophilus* B5 isolated from compost reveal its enzymatic basis for lignocellulose degradation. Microorganisms 8:1357. doi: 10.3390/microorganisms8091357, 32899798 PMC7564440

[ref42] Wang B. Zhang P. Guo X. Bao X. Tian J. Li G. . (2024). Contribution of zeolite to nitrogen retention in chicken manure and straw compost: reduction of NH3 and N2O emissions and increase of nitrate. Bioresour. Technol. 391:129981. doi: 10.1016/j.biortech.2023.129981, 37926358

[ref43] Wei S. Li Z. Sun Y. Zhang J. Ge Y. Li Z. (2022). A comprehensive review on biomass humification: recent advances in pathways, challenges, new applications, and perspectives. Renew. Sust. Energ. Rev. 170:112984. doi: 10.1016/j.rser.2022.112984

[ref44] Xin L. Qin Y. Lou T. Xu X. Wang H. Mei Q. . (2023). Rapid start-up and humification of kitchen waste composting by an innovative biodrying-enhanced process. Chem. Eng. J. 452:139459. doi: 10.1016/j.cej.2022.139459

[ref45] Xiong Y. (2022). Characterization and variation of dissolved organic matter in composting: a critical review. Front. Environ. Sci. Eng. 17:63. doi: 10.1007/s11783-023-1663-7

[ref46] Xu Z. Tsang D. C. (2024). Mineral-mediated stability of organic carbon in soil and relevant interaction mechanisms. Eco-Environ. Health. 3, 59–76. doi: 10.1016/j.eehl.2023.12.003, 38318344 PMC10840363

[ref9001] Yang Y. Kong Y. Wang G. Shen Y. Tang R. Yin Z. . (2023). Temporal succession and spatial heterogeneity of humification, pathogens and bacterial community in facultative heap composting. Process Safety and Environmental Protection 176, 734–746., 38543636

[ref47] Yu Z. Wang B. Wu X. Yu R. Shen L. Wu X. . (2024). Exploration of the vermiculite-induced bacterial community and co-network successions during sludge–waste mushroom co-composting. Microorganisms 12:585. doi: 10.3390/microorganisms12030585, 38543636 PMC10976085

[ref48] Zhan Y. Chang Y. Tao Y. Zhang H. Lin Y. Deng J. . (2023). Insight into the dynamic microbial community and core bacteria in composting from different sources by advanced bioinformatics methods. Environ. Sci. Pollut. Res. 30, 8956–8966. doi: 10.1007/s11356-022-20388-7, 35462586

[ref49] Zhang Y. Chen M. Guo J. Liu N. Yi W. Yuan Z. . (2022). Study on dynamic changes of microbial community and lignocellulose transformation mechanism during green waste composting. Eng. Life Sci. 22, 376–390. doi: 10.1002/elsc.202100102, 35573133 PMC9077819

[ref50] Zhang L. Sun X. (2017). Addition of seaweed and bentonite accelerates the two-stage composting of green waste. Bioresour. Technol. 243, 154–162. doi: 10.1016/j.biortech.2017.06.099, 28654836

[ref51] Zhang S. Wang L. Zhou B. Zhang D. Tang G. Guo L. (2024). Characteristics of humification, functional enzymes and bacterial community metabolism during manganese dioxide-added composting of municipal sludge. Environ. Res. 252:119151. doi: 10.1016/j.envres.2024.119151, 38754608

[ref52] Zhao Y. Zhang W. Wang S. Liu J. Li Y. Zhuo Y. (2020). Effects of soil moisture on the reclamation of sodic soil by flue gas desulfurization gypsum. Geoderma 375:114485. doi: 10.1016/j.geoderma.2020.114485

[ref53] Zheng W. Yang Z. Huang L. Chen Y. (2022). Roles of organic matter transformation in the bioavailability of cu and Zn during sepiolite-amended pig manure composting. J. Environ. Manag. 314:115046. doi: 10.1016/j.jenvman.2022.115046, 35468432

[ref54] Zhou L. Yang X. Wang X. Feng L. Wang Z. Dai J. . (2023). Effects of bacterial inoculation on lignocellulose degradation and microbial properties during cow dung composting. Bioengineered 14, 213–228. doi: 10.1080/21655979.2023.2185945, 37471462 PMC10599258

[ref55] Zhu N. Gao J. Liang D. Zhu Y. Li B. Jin H. (2020). Thermal pretreatment enhances the degradation and humification of lignocellulose by stimulating thermophilic bacteria during dairy manure composting. Bioresour. Technol. 319:124149. doi: 10.1016/j.biortech.2020.124149, 32979596

[ref56] Zhu P. Li Y. Gao Y. Yin M. Wu Y. Liu L. . (2021). Insight into the effect of nitrogen-rich substrates on the community structure and the co-occurrence network of thermophiles during lignocellulose-based composting. Bioresour. Technol. 319:124111. doi: 10.1016/j.biortech.2020.124111, 32971335

