# Supplementary material for: Mineral-specific effects on humification and bacterial community succession in industrial-scale spent mushroom substrate composting
Source: Front Microbiol. 2026 Sep 4;17:1952248. doi: 10.3389/fmicb.2026.1952248 (PMC13585754; doi:10.3389/fmicb.2026.1952248)
Supplement: Supplementary file 1 [file Supplementary_file_1.docx]

***Supplementary Materials***

**Mineral-specific effects on humification and bacterial community succession in industrial-scale spent mushroom substrate composting**

**Content**

[Table S1 Basic properties of raw materials	1](#_Toc26670)

[Table S2 The basic properties of initial mixtures	2](#_Toc4533)

[Table S3 Specific surface area, pore volume, and average pore diameter of the different minerals	3](#_Toc22783)

[Table S4 Humification indices, proportions of DOM components, and their statistical significance among different treatments during composting	4](#_Toc10864)

[Table S5 Topological indices of bacterial network in different mineral-amended composting	6](#_Toc10079)

[Figure. S1 N_2_ physical adsorption-desorption isotherms of different minerals	7](#_Toc26434)

[Figure. S2 Changes in the three-dimensional excitation-emission matrix fluorescence spectroscopy of DOM during composting	8](#_Toc9272)

[Figure. S3 Changes in the structure of parallel factor components of DOM during composting	9](#_Toc21864)

[Fig. S4 Correlation matrix between mineral physicochemical properties and composting outcomes. Spearman correlations were calculated between mineral structural properties and selected physicochemical, humification, and DOM-related indicators. Red and blue denote positive and negative correlations, respectively	10](#_Toc12236)

[Figure. S5 Bray-Curtis dissimilarity between mineral-amended treatments and the control across composting phases	11](#_Toc9268)

[Figure. S6 Bacterial co-occurrence networks under different mineral treatments. Nodes represent bacterial OTUs; Node colors denote major network modules, whereas gray nodes represent taxa belonging to the remaining modules; Edge colors distinguish positive and negative associations; red and green edges indicate positive and negative correlations, respectively	12](#_Toc5110)

Table S1 Basic properties of raw materials

| Raw materials | Moisture content/% | pH value | Electric conductivity/mS·cm^-1^ | Total Organic carbon/% | Total nitrogen/% |
| --- | --- | --- | --- | --- | --- |
| Spent mushroom substrate | 60.10±0.70 | 5.26±0.05 | 4.82±0.10 | 32.33±1.57 | 1.51±0.29 |
| Shale | 12.96±0.37 | 9.02±0.63 | 0.20±1.53 | — | — |
| Mullite-based soil conditioner | 13.14±0.34 | 11.75±0.16 | 4.48±0.03 | — | — |
| Desulfurization gypsum | 12.50±2.77 | 9.11±0.08 | 4.02±0.16 | — | — |
| Rare earth silicon titanium ore powder | 1.13±0.30 | 12.97±0.04 | 6.32±0.06 | — | — |

Table S2 The basic properties of initial mixtures

| Treatment | Moisture content/% | pH value | Electric conductivity/mS·cm^-1^ | Total Organic carbon/% |
| --- | --- | --- | --- | --- |
| CK | 60.1±0.7 | 5.30±0.05 | 4.80±0.10 | 32.3±1.6 |
| S | 56.2±0.2 | 6.40±0.03 | 4.10±0.09 | 28.5±0.5 |
| SC | 55.9±0.6 | 7.20±0.04 | 4.30±0.06 | 29.9±0.2 |
| DG | 55.9±0.3 | 5.70±0.23 | 6.00±0.16 | 32.3±0.1 |
| TO | 57.3±0.4 | 5.50±0.02 | 4.80±0.01 | 31.0±0.6 |

Table S3 Specific surface area, pore volume, and average pore diameter of the different minerals

| Mineral | | Specific surface area (m^2^/g) | Total pore volume (cm^3^/g) | Micropore volume (cm^3^/g) | Average pore diameter (nm) |
| --- | --- | --- | --- | --- | --- |
| Shale | 9.8173 | | 0.0097 | 0.0019 | 12.6811 |
| Mullite-based soil conditioner | 9.6536 | | 0.0201 | 0.0021 | 28.9875 |
| Desulfurization gypsum | 8.0965 | | 0.0208 | 0.0019 | 29.8818 |
| Rare earth silicon titanium ore powder | 3.5255 | | 0.0044 | 0.0009 | 18.6398 |

Table S4 Humification indices, proportions of DOM components, and their statistical significance among different treatments during composting

| Phase | Treatment | HS  (mg/g) | HA  (mg/g) | FA  (mg/g) | DP | HR  (%) | HI  (%) | DOM-C1  (%) | DOM-C2  (%) | DOM-C3  (%) |
| --- | --- | --- | --- | --- | --- | --- | --- | --- | --- | --- |
| H | CK | 7.48±0.63 b | 5.32±0.53 b | 2.16±0.10 bc | 2.46±0.33 bc | 2.29±0.31 a | 1.63±0.63 a | 30.78±0.62 c | 49.52±1.42 a | 19.70±0.55 bc |
|  | S | 8.90±0.40 a | 6.82±0.36 a | 2.08±0.04 c | 3.28±0.36 ab | 3.10±0.40 a | 2.38±0.40 a | 38.21±0.83 b | 43.12±0.89 b | 18.67±0.73 cd |
|  | SC | 9.30±0.33 a | 7.19±0.29 a | 2.11±0.04 c | 3.41±0.29 a | 3.13±0.41 a | 2.42±0.33 a | 31.93±1.05 c | 44.66±1.36 bc | 23.41±1.38 a |
|  | DG | 9.07±0.48 a | 6.53±0.41 a | 2.54±0.06 a | 2.57±0.41 abc | 3.09±0.64 a | 2.22±0.48 a | 30.61±0.73 c | 47.05±1.11 ac | 22.34±1.91 ab |
|  | TO | 7.23±0.38 b | 4.94±0.32 b | 2.29±0.06 b | 2.16±0.32 c | 2.51±0.66 a | 1.71±0.38 a | 44.43±1.11 a | 39.30±1.94 d | 16.27±0.67 d |
| T | CK | 11.85±0.47 b | 7.65±0.32 c | 4.20±0.15 ab | 1.82±0.32 bc | 3.93±0.50 ab | 2.54±0.47 ab | 45.73±1.26 a | 38.11±0.34 c | 16.16±0.42 c |
|  | S | 8.76±0.18 c | 4.74±0.15 e | 4.02±0.03 b | 1.18±0.15 c | 3.20±0.32 b | 1.73±0.18 b | 38.79±0.88 bc | 42.72±0.84 a | 18.50±0.33 b |
|  | SC | 10.99±0.27 b | 6.82±0.20 d | 4.17±0.08 ab | 1.64±0.20 bc | 4.00±0.81 ab | 2.48±0.27 ab | 37.92±1.31 c | 37.90±0.72 c | 24.18±0.28 a |
|  | DG | 12.96±0.49 a | 8.64±0.35 b | 4.32±0.14 a | 2.00±0.35 b | 4.90±0.42 a | 3.27±0.49 a | 43.17±2.83 ab | 39.95±0.27 b | 16.88±0.16 c |
|  | TO | 12.92±0.28 a | 9.49±0.25 a | 3.43±0.03 c | 2.77±0.25 a | 4.62±0.33 a | 3.39±0.28 a | 44.98±1.99 a | 38.69±0.36 bc | 16.33±0.37 c |
| C | CK | 12.53±0.31 b | 8.64±0.17 b | 3.89±0.14 b | 2.22±0.17 bc | 3.94±0.44 b | 2.71±0.31 b | 38.45±1.09 a | 41.85±0.22 c | 19.70±0.23 d |
|  | S | 10.83±0.20 c | 6.84±0.20 c | 3.99±0 ab | 1.71±0.20 c | 3.59±0.30 b | 2.27±0.20 b | 31.32±0.52 b | 45.49±0.96 b | 23.19±0.55 c |
|  | SC | 12.87±0.04 b | 8.72±0.02 b | 4.15±0.02 a | 2.10±0.02 bc | 4.20±0.20 b | 2.84±0.04 b | 28.35±0.21 c | 41.11±0.81 c | 30.54±0.05 b |
|  | DG | 12.49±0.42 b | 9.01±0.37 b | 3.48±0.05 c | 2.59±0.37 ab | 4.13±0.53 b | 2.98±0.42 b | 26.29±0.11 d | 41.98±0.72 c | 31.73±0.49 a |
|  | TO | 14.48±0.19 a | 10.88±0.17 a | 3.60±0.02 c | 3.02±0.17 a | 5.28±0.22 a | 3.97±0.19 a | 28.46±0.57 c | 47.98±0.89 a | 23.56±0.36 c |
| M | CK | 13.41±0.25 d | 9.89±0.12 c | 3.52±0.13 a | 2.81±0.12 c | 5.63±0.30 b | 4.15±0.25 c | 30.11±0.87 a | 35.09±0.65 c | 34.80±0.08 c |
|  | S | 15.34±0.75 ab | 11.93±0.71 a | 3.41±0.03 a | 3.50±0.21 ab | 6.72±0.30 ab | 5.23±0.75 ab | 26.20±0.28 b | 35.84±0.13bc | 37.97±0.51 b |
|  | SC | 16.05±0.06 a | 12.67±0.03 a | 3.38±0.09 a | 3.75±0.03 a | 7.45±0.90 a | 5.88±0.06 a | 17.26±0.57 c | 41.93±0.17 a | 40.81±0.28 a |
|  | DG | 14.47±0.21 bc | 10.95±0.23 b | 3.52±0.02 a | 3.11±0.23 bc | 6.31±0.40 ab | 4.77±0.21 bc | 28.98±0.22 a | 41.67±0.38 a | 29.34±0.23 d |
|  | TO | 14.12±0.09 cd | 10.67±0.18 bc | 3.45±0.09 a | 3.09±0.18 bc | 6.37±0.90 ab | 4.82±0.09 bc | 29.69±0.75 a | 36.31±0.24 b | 34.00±0.44 c |

Note: Different lowercase letters represent significant difference between treatments (*P* < 0.05).

Table S5 Topological indices of bacterial network in different mineral-amended composting

| Network specificity | CK | S | SC | DG | TO |
| --- | --- | --- | --- | --- | --- |
| Number of nodes | 167 | 170 | 134 | 138 | 100 |
| Number of edges | 1431 | 1152 | 548 | 815 | 520 |
| Average degree | 17.138 | 13.553 | 8.179 | 5.906 | 10.4 |
| Network density | 0.103 | 0.080 | 0.061 | 0.086 | 0.105 |
| Modularization | 0.674 | 0.842 | 0.903 | 0.829 | 0.748 |
| Positive correlation (%) | 88.12 | 70.40 | 71.53 | 61.53 | 74.04 |
| Negative correlation (%) | 11.88 | 29.60 | 28.47 | 38.65 | 25.96 |

Figure. S1 N_2_ physical adsorption-desorption isotherms of different minerals

Figure. S2 Changes in the three-dimensional excitation-emission matrix fluorescence spectroscopy of DOM during composting

Figure. S3 Changes in the structure of parallel factor components of DOM during composting

Fig. S4 Correlation matrix between mineral physicochemical properties and composting outcomes. Spearman correlations were calculated between mineral structural properties and selected physicochemical, humification, and DOM-related indicators. Red and blue denote positive and negative correlations, respectively

Figure. S5 Bray-Curtis dissimilarity between mineral-amended treatments and the control across composting phases

Figure. S6 Bacterial co-occurrence networks under different mineral treatments. Nodes represent bacterial OTUs; Node colors denote major network modules, whereas gray nodes represent taxa belonging to the remaining modules; Edge colors distinguish positive and negative associations; red and green edges indicate positive and negative correlations, respectively
